# The influence of community structure on opinion expression: an agent-based model

**DOI:** 10.1007/s11573-021-01064-7

**Published:** 2021-10-20

**Authors:** Benjamin Cabrera, Björn Ross, Daniel Röchert, Felix Brünker, Stefan Stieglitz

**Affiliations:** University of Duisburg-Essen, Duisburg, Germany

**Keywords:** Agent-based model, Spiral of silence, Network, Communities, Stochastic block model, C15, D85

## Abstract

Social media has become important in shaping the public discourse on controversial topics. Many businesses therefore monitor different social media channels and try to react adequately to a potentially harmful opinion climate. Still, little is known about how opinions form in an increasingly connected world. The spiral of silence theory provides a way of explaining deviations between the perceived opinion climate and true beliefs of the public. However, the emergence of a spiral of silence on social media is hard to observe because only the thoughts of those who express their opinions are evident there. Recent research has therefore focused on modelling the processes behind the spiral of silence. A particular characteristic of social media networks is the presence of communities. Members of a community tend to be connected more with other members of the same community than with outsiders. Naturally, this might affect the development of public opinion. In the present article we investigate how the number of communities in a network and connectivity between them affects the perceived opinion climate. We find that higher connectivity between communities makes it more likely for a global spiral of silence to appear. Moreover, a network fragmented into more, smaller communities seems to provide more “safe spaces” for a minority opinion to prevail.

## Introduction

Businesses are influenced in many ways by the discussions that take place on social media. Sometimes, these take the form of short-lived social media storms that may threaten a company’s reputation, for example in the case of a faulty product or claims of employee abuse, but they can also blow over quickly. At other times, these discussions can have serious long-term implications for individual businesses or for entire industries, with potential ripple effects throughout their supply chains. Current examples include debates on the carbon emissions of the various forms of private and public transport, such as cars and aeroplanes. These debates on social media do not only reflect the social norms around the usage of specific modes of transport, but they also shape them.

In order to make informed strategic decisions, it is essential for companies to understand the underlying processes shaping public opinion. One specific aspect of these processes is described by the spiral of silence theory (Noelle-Neumann 1974). It applies to controversial issues on which there are two opposing viewpoints, and it assumes that people’s willingness to express their opinion (for example, for or against a new policy) depends on the opinions expressed by those around them. If they sense that their peers agree with them, they become more likely to voice their own opinion and the reverse. Over time, the theory posits, this behaviour can spiral into a situation in which a clear majority of publicly expressed opinions are in favour of one of the two viewpoints, and a consensus is established. A central point of the spiral of silence theory is that this apparent consensus opinion does not actually need to be held by the majority. The minority that espouses it might simply be more confident in their opinion and therefore more vocal.

This theory has been shown to apply to social media settings, to some extent. Recent simulation approaches show how individual decisions to spread one’s opinion or not translate, on a macro level, into group dynamics that establish social norms (Ross et al. 2019). However, a key aspect of social media communication that has not been addressed in previous research, is that it often takes place in communities which are sometimes well-connected to other communities, but might also be more or less isolated. A real-life example of this would be a company that sells its products in various geographic markets, or to different segments of the population. This paper, therefore, explores how the development of a (perceived) public opinion, following the spiral of silence theory, is affected when the network is more or less subdivided into communities.

To form a better view of the spiral of silence process within and between online communities, we developed a new network model that is able to express various forms of community structure, and otherwise applied the same agent behaviour, regarding the spiral of silence, as in the work published by Ross et al. (2019). We use this model to investigate the influence of two key parameters: the size and number of communities (that is, whether the overall network is divided into many small ones or into few large ones), and the interconnectedness of the communities (that is, how many edges there are between communities, relative to how many there could be). In network terminology, the first aspect relates to the distribution of nodes, the second to the distribution of edges. In the business context, the first question reflects how segmented the market is, while the second question reflects how close the markets or segments are to each other.

The paper makes the following contributions to the literature. Compared to previous studies on the spiral of silence, it clarifies how community structure affects this process. The more fragmented the network is into smaller communities, the less likely it is that one opinion is silenced entirely. The more interconnected the network, the more likely a global spiral of silence is to emerge in which only the supporters of one opinion are willing to express it. While previous studies on opinion dynamics in social networks that show community structure came to similar conclusions, these other studies were not based on the spiral of silence theory. Our work therefore shows that the assumptions of the spiral of silence can serve as an alternative explanation for this phenomenon. At the same time, it demonstrates the usefulness of agent-based modelling as a simulation technique.

The following section explains the necessary background information on how online opinion formation may impact business success, the role community structure plays in online social networks, and the spiral of silence theory. Section 3 describes the method, that is, the agent-based model and how it was validated. The results are shown in Sect. 4 and discussed in Sect. 5, with emphasis on their implications for research and for businesses.

## Background

### The impact of public opinion formation online on business success

In order to reduce uncertainty about their own decisions, customers often rely on information shared by others (Bikhchandani et al. 1992). Social media enhances the information exchange of product information and ratings by providing low-cost functionalities to reach a large audience and easily establish connections to other people. Thus, the formation of public opinion online has become even more important in the context of business success.

Research suggests that marketing managers observe public discourse to identify users’ complaints and needs. This might lead to an improvement in the company’s image. Likewise, the enhancement of a brand image could be due to the fact that the feelings and needs of customers are perceived and taken into account by the company's decision-makers (Kaiser et al. 2011). However, it is crucial to detect upcoming negative opinions towards a product, brand or person before they spread in the network or community. The detection and counteraction are particularly important due to the finding that customers’ opinions could be influenced by the opinions of others (Sunder et al. 2019). In general, customers adapt their reviews or ratings of products to the general opinion of the crowd (Muchnik et al. 2013; Jiang and Wu 2017). Furthermore, if two reference groups have distinct opinions, experienced users rely more on their friends’ opinions than on the crowd. In contrast, new or inexperienced users, who have had less time to establish strong connections to others, rely more on the crowd than on new friends on social media (Sunder et al. 2019). In this context, people might weigh their connection to others such as the crowd, media outlets, or opinion leaders, that is, users who are likely to influence other users within their personal network (Jiang and Wu 2017; Watts and Dodds 2007; Katz and Lazarsfeld 1955).

### Community structure in online social networks

The possibility to get in contact with people across temporal and spatial distances and to communicate with them is omnipresent. Social online platforms such as Twitter, Facebook or YouTube provide opportunities for networking and community formation. In this context, the term community describes a group of nodes (that is, users or accounts) that are more strongly connected to each other than to the rest of the network.

The investigation of political communities in terms of their participation has already been the goal of many studies (Grace-Farfaglia et al. 2006; Oser et al. 2013; Velasquez 2012). Similarly, studies found that national cultures also differ in their active participation in online political communities, according to the findings, 13.7% of Americans participate in political communities, compared to 7.45% in the Netherlands and 6.1% in South Korea (Grace-Farfaglia et al. 2006).

In addition, there are various reasons and motives for people to join communities, e.g., information and social friendship building (Ridings et al. 2006) or gaining a deeper understanding of the opinions and attitudes of others (Herring 1996). The exchange of political or ideological opinions, in which the individual’s point of view is reinforced, can lead to the emergence of virtually homogeneous spaces, so-called “echo chambers”, wherein like-minded people interact *only* with each other (Boutyline and Willer 2017). It is also argued that such self-reinforcing “echo chambers” can be seen as a danger to society, as they are particularly associated with polarisation and radicalisation because users are more extreme in their views (Prior 2007). Seen in this way, homophily, that is, “the principle that a contact between similar people occurs at a higher rate than among dissimilar people” (McPherson et al. 2001, p. 416), could also be a reason for the formation of communities.

Political or opinion-based homogeneous discussion areas have already been examined on Facebook (Bakshy et al. 2015), YouTube (Röchert et al. 2020) and Twitter (Barberá et al. 2015), but the aspect of the individual communities within the network has largely been ignored. Williams et al. (2015) analysed the Twitter communication network on climate change using a network analysis and found that there is a strong homogeneity in the interactions between like-minded communities of climate change activists and climate sceptics. More specifically, climate change activists expressed positive opinions with each other, while climate sceptics expressed negative opinions among themselves. Furthermore, the authors were able to identify mixed communities, which were characterized by a balanced and polarized content. The results of homogeneous communities are in line with the results of Conover et al. (2011), who found that the retweet network for political communication on Twitter during the 2010 U.S. midterm elections was very polarized, with only few connections between left- and right-leaning users.

### The spiral of silence

The spiral of silence theory (Noelle-Neumann 1974) explains changes in people’s willingness to express their opinion as the result of a fear of being socially isolated. People sense the opinions on controversial topics of those around them and modify their public behaviour accordingly. Over time, this results in the formation of a consensus, the establishment of a social norm. Crucially, this consensus opinion does not even need to be held by the majority. It could simply be the case that the minority that holds this opinion is especially vocal about it, or is using especially effective communication channels to reach many people, leading the actual majority to not express their opinion openly. The assumptions of this theory have been the subject of much empirical research. In the realm of social media, it has been shown that individuals are (slightly) affected in their assessment of the overall opinion distribution by what they see online (Neubaum and Krämer 2017).

Although it has frequently been applied to attitudes towards political questions such as capital punishment, the spiral of silence theory in the original conceptualisation has always applied to a wide range of social norms including, for example, homeowners shovelling snow from their share of the sidewalk (Noelle-Neumann 1974). If they affect consumption decisions, the social pressures and group norms explained by this theory can have long-term strategic implications for businesses and entire industries.

A recent example is the Swedish concept of “flygskam” (flying shame or flight shame). Aware of the impact of air travel on carbon emissions, the environmentally conscious switch to other modes of transport (Weston et al. 2019). This choice is often communicated publicly, for example on social media. Climate activist Greta Thunberg’s decision to sail to the UN climate summit by yacht instead of flying was widely and controversially discussed in traditional and social media (Parker, 2019). Although empirical evidence does not yet indicate that those with a higher awareness of climate change have lower greenhouse gas emissions from flights—if anything, the opposite is the case (Czepkiewicz et al. 2019)—and although its reach is geographically limited, with many markets for air travel, such as the Asia–Pacific Region, experiencing unprecedented growth (IATA 2018), if this movement grows it might threaten air travel as a leisure activity. According to news reports, airline executives were already worried in 2019 (Rucinski et al. 2019). In 2020–2021, the COVID-19 pandemic severely hit the airline industry. Although at the time of writing, its permanent effects are still hard to predict, it seems likely that some peer groups will exert additional social pressure against long-distance travel to avoid spreading the disease.

A related example is that of choosing to drive a car and choosing which car to drive. In a study by Hopkins (2016) in New Zealand, the Generation Y interviewees were highly aware of the environmental impact of cars, especially to commute, and some participants decided not to drive for environmental reasons despite owning a driver’s license.

A guilty conscience due to the perceived negative effects of transport choice would not in itself be enough to meet the theoretical assumptions of the spiral of silence. The theory does not predict changes in people’s privately held opinions, but in the expressions of these opinions. The individuals in question would need to fear being socially isolated as a result of their choices. Indeed, previous research has at least surmised a link between social status and environmentally conscious consumption choices. Kahn (2007) showed a difference between environmentally indifferent “brown” communities and green ones where “the group norm is to live a sustainable lifestyle … driving a Prius would increase one’s status while driving a Hummer would have the opposite effect”. In making this link, we assume that the communication of one’s opinion does not necessarily need to happen verbally, since the choice to buy and drive a car is an equally public display of one’s attitudes.

### Related work on simulating opinion dynamics

Complex communication processes such as the spiral of silence are challenging to investigate due to the complexity of empirical test procedures, as they require many resources, such as long-term observations of experiments and also the need for a large number of participants (Waldherr and Wettstein 2019). With the help of agent-based modelling, it is possible to investigate social phenomena of micro-level findings at the macro level (Epstein 2006; Klein et al. 2018), such as the dynamic processes within a network and how the interactions within the agents develop (Bruch and Atwell 2015).

In the literature on opinion dynamics, a variety of different methods based on mathematical and physical rules exist to simulate the mechanisms of interaction and their influence on opinions (Castellano et al. 2009). However, because there are many different modelling decisions to make, research questions to answer and results to focus on, a comprehensive overview of the field of opinion dynamics would be out of scope here. The following paragraphs, therefore, each focus on a different aspect of the research, namely the different types of interactions between opinions used in different models, the effect of communities and existing models studying the spiral of silence.

Models simulating opinion dynamics can broadly be split into those modelling opinions as discrete values [the Voter model (Clifford and Sudbury 1973; Holley and Liggett 1975), Snajzd model (Sznajd-Weron and Sznajd 2000)] and those using some form of continuous representation of opinions. The main difference between these two categories is the range of opinions that the agents in the models can assume. Discrete models often have binary opinions (i.e., in favour, against), while in continuous models, opinions are scalars, often bounded by an interval of values.

A well-studied class of continuous models are so-called bounded confidence models (Deffuant et al. 2000; Hegselmann and Krause 2002). These models consist of a set of agents, each of which is assigned an opinion, modelled as a real value in the interval [0,1]. The difference between these models lies in their view of how they implement the communication between individuals. In the Deffuant model, the dynamic is based on the interaction of two individuals randomly connected to each other in the network, while the HK model considers the interactions of individuals in larger groups. The opinion values change over time depending on the values of the other agents, and the strength of the connections between the agents. The non-linearity and “boundedness” of the models is introduced by the fact that an agent only considers opinions that deviate up to a bound from its own opinion value. Interestingly, depending on the initial configuration and model parameters, the formation of clusters of agents with similar opinions can be observed (Lorenz 2006). Another classical continuous model, the DeGroot model, can be classified in the category of averaging models, in which agents determine their opinion based on the average of their neighbours' opinions (DeGroot 1974). This model has been used as the foundation for other models, such as the Friedkin-Johnsen model, which takes into account the aspect of stubbornness, so that individuals hold on to their original opinion to a certain level (Friedkin and Johnsen 1990). In a study in the area of interpersonal social influence, Ye et al. (2019) explicitly distinguish between private and expressed opinions in order to identify how they can deviate from another over time. Here, they used a strongly connected, aperiodic directed network to show that the combination of the network's strong interconnectedness, the individual’s pressure to conform, and the individual’s stubbornness have an impact on the discrepancy.

While the previously mentioned articles focus mainly on the different ways opinion interaction can be modelled, other research modelling opinion dynamics has specifically focused on studying the effects of community structure. The non-linear model of Banisch and Olbrich (2019), based on reinforcement learning (Q-learning), addresses the question of how bi-polarised opinion distributions can emerge and persist. In their model, the individual agents learn of and adapt to the opinions of their neighbours. To model the social structure, a random geometric graph was used in which agents communicate with those they are physically close to. The presence of communities and structural holes is a key aspect that allows the formation of a stable polarised opinion climate: in dense, less modular networks, polarisation disappears in favour of a global consensus. In a recent study by Stern and Livan (2021), the DeGroot and Friedkin-Johnsen models were used and extended to investigate the diversity of opinions in networks. Here, the network structure used was a stochastic block model; their results showed that the diversity of opinions decreases due to closed communities and thus it is more difficult to come to a common consensus.

There are already models specifically focussed on simulating the circumstances around the spiral of silence theory. In an article by Wu et al. (2015), an agent-based model is proposed where each agent is initialized holding one of two opinions. Then, a single agent is selected (the “first speaker”) who expresses its opinion and triggers its neighbours to either also express their opinion, or stay silenced, depending on an “opinion pressure” which is based on the network topology around the agents and their neighbours opinions. Agents that have either expressed their opinion or stayed silent become “immune” and can’t be triggered again. This process continues until no agents are left that could trigger a response. This model was then used to study the global opinion distribution based on different network topologies. The spiral of silence was also examined in a setting of more complex agent behaviour by Sohn and Geidner (2016). Here, the agents randomly move around on a two-dimensional plane and express their opinion (if they are confident enough to do so) only to those agents that are physically close to them. In the model by Ross et al. (2019), inspired by the agent behaviour used by Sohn and Geidner (2016), the agents' influence is determined by a small-world, scale-free network that is more representative of online social networks and a world with internet-based communication. A recent study by Ma and Zhang (2021) used agent-based simulation for a model of opinion expression dynamics inspired by the spiral of silence theory in that people’s willingness to express their opinion depends on perceived peer support for that opinion. Since their goal was to model a social media chat group where every user sees every other user’s posts, their simulation assumed a fully connected network.

The spiral of silence networks studied by Sohn and Geidner (2016), Ross et al. (2019), and Ma and Zhang (2021) do not exhibit a community structure. However, real social networks exhibit varying amounts of modularity (Guerra et al. 2013), and a good simulation model for the discussed cases should therefore directly take into account community structure.

## Methods

To test the effects of community structure on the spiral of silence process, we used an agent-based simulation model. Agent-based models are used in a variety of disciplines, from physics and biology to the social sciences (Wilensky and Rand 2015). Their strength lies in their versatility. The user specifies an environment and agents that populate it, including the rules according to which the individual agents act and react to their surroundings. It is then possible to observe the model as time passes, to pause and inspect the model and to calculate various statistics at any point in time.

This simulation approach has various advantages over other approaches that study social media usage. The simulation can be carried out an arbitrary number of times and populated with an arbitrary number of agents, unlike laboratory experiments, which are unfeasible for opinion formation processes in very large groups. Quasi-experimental studies in which the behaviour of many is manipulated are morally questionable (Flick 2016). Finally, unlike in large-scale observational studies, it is possible to examine the variables of agents, that is, to peer into the minds of those who remain silent. Otherwise, if messages such as Facebook posts and tweets are examined, the study would be limited to examining the opinions of those who are willing to express them. However, a critical challenge when working with agent-based models is to ensure that they accurately reflect reality. This section describes the network model, agent behaviour, and validation measures taken.

The simulation model is largely based on Ross et al. (2019)’s. The key difference is that the network model includes subcommunities. We also reimplemented the model in C++, as this allows for much faster run times and more flexibility compared to the original NetLogo implementation. The ability to perform more simulation runs translates into an increased precision of results. The source code is freely available on GitHub https://github.com/bencabrera/spiral_of_silence_abm.

### Modelling networks with cohesive communities

Networks are at the heart of our agent-based model for simulating the spiral of silence. They define the interaction topology of the agents by determining which agents another agent considers when gauging the opinion climate. Since the process of producing results from an agent-based model involves averaging outcomes of many simulations, a network model is needed to randomly generate new instances to run the simulations on.

This work differs from Ross et al. (2019) in the network model used. Ross et al. (2019) used a preferential attachment model (Albert and Barabási 2002), as it creates power-law tailed degree distributions typical for social networks (Barabási and Albert 1999) and is often considered a good, albeit simple, model of social networks in general (Newman 2003). The focus of this work, in contrast, lies on studying the effects of network communities on the spiral of silence. It is therefore essential to have a method of reliably generating networks containing ground-truth communities, which the simple preferential attachment models are not capable of.

The following paragraphs contain a brief review of important definitions. First of all, we only consider undirected networks, formally characterized by the mathematical notion of an undirected graph $G = \left( {V,E} \right)$, where $V$ is a set of agents (or nodes) and $E \subset \left\{ {\left\{ {u,v} \right\} : u, v \in V} \right\}$ the set of connections (or edges). This implies that any influence between two agents runs both ways. If agent A and agent B are connected by an edge, then agent A is influencing B as well as the other way around. The *density ρ* of a network is the number of edges in the network divided by the number of possible edges, i.e. $\rho = \frac{2\left| E \right|}{{\left| V \right|\left( {\left| V \right| - 1} \right)}}$.

A community structure in a network (Wasserman and Faust 1994) can be described by partitioning the nodes into multiple subsets $V_{i} \subset V$ (the communities) according to some characteristics. While Wasserman and Faust (1994) propose different such characteristics, the one most commonly used in empirical network analysis (Fortunato 2010) is based on the idea that network communities ought to have more connections between members of the same community (*intra-community edges*) than between members of different communities (*inter-community edges*). Analogue to the density, one can also define the *intra-community density* $\rho_{in}$ and the *inter-community density* $\rho_{out}$ as the ratios between the number of intra-/inter-community edges and the number of all possible edges of the respective type. With these definitions in place, we say a node partition actually represents a community structure if $\rho_{in}$ is significantly higher than $\rho_{out}$, with the meaning of “significantly” depending on the actual case at hand. With the notion of network communities defined, it is still unclear how networks with such communities can be generated reliably.

The most common type of network models used to generate networks with communities is stochastic block models (SBM). Different variants of stochastic block models have been proposed (Abbe 2017). However, generally, they take at least three parameters: the total number of nodes $n$, a partition of the node set into $r$ communities, and a $r \times r$ matrix $P$ of probabilities, where $P_{i,j}$ specifies the probability of connecting nodes from community $i$ with nodes from community $j$. Sometimes the partition into communities is also sampled from a given probability distribution (Abbe 2017). The network is then built by randomly deciding for every pair of vertices independently if the two vertices should be connected by an edge or not, using the $P$ matrix entries as explained before. Note that the diagonal values $P_{i,i}$ characterise the probability of connecting two nodes inside the same community $i$, while the $P_{i,j}$ for $i \ne j$ are the probabilities of connecting nodes of different communities. This implies that the diagonal values of $P$ are typically chosen much larger than the off-diagonal ones, in order to create cohesive communities. Also, for a constant probability matrix $P_{i,j} = c$ for all $1 < = i,j < = r$, the model is equal to an Erdös-Renyi model (Erdős and Rényi 1959) with parameter $c$, and therefore no community structure would be visible.

This classic Stochastic Block Model has several drawbacks that lead us to use a slightly different model for generating networks. First, networks generated from a classic SBM do not exhibit a power-law tailed degree distribution, typical for social networks. Instead, in the classic SBM every community is essentially an Erdös–Renyi random graph that has a Poisson distribution of the degrees (Newman 2003). An even bigger problem with a classic SBM is the fact that the generated networks are not necessarily connected. Especially for the targeted densities, Erdös-Renyi graphs tend to break down into many disconnected components, which is not desired in an agent-based model that relies on connections for propagating influence to other agents.

**Fig. 1:**
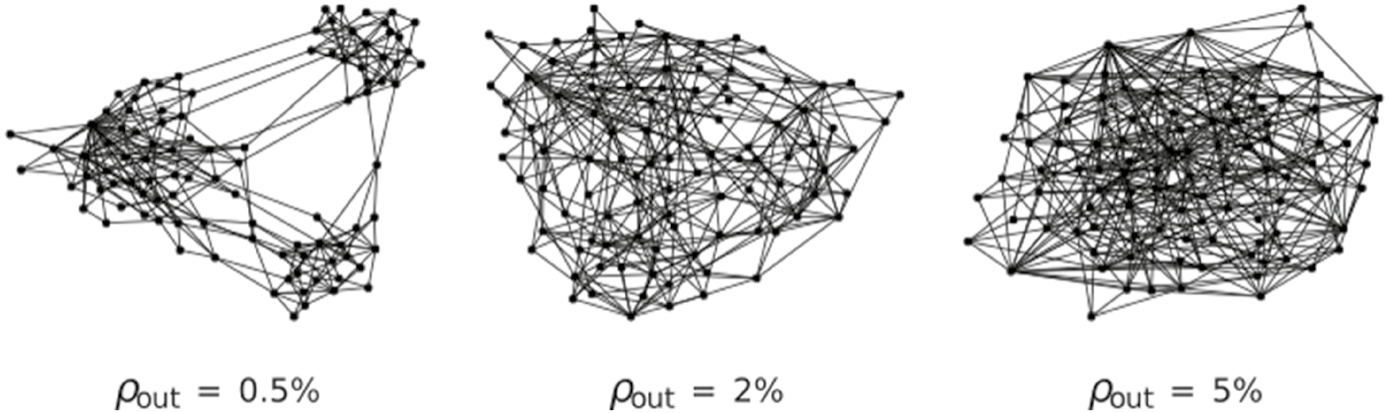
Three networks generated with the stochastic block model used in the simulations. All networks have 100 nodes and an a-priori (50, 25, 25) partition into communities. Every community is generated by the Barabási-Albert model with $m = 3$. From left to right, higher inter-community densities $\rho_{out}$ are used. In the left drawing, the three communities are clearly visible. The network in the middle already has many inter-community edges, such that the communities start to blend and in the rightmost drawing almost no communities are visible

To solve these problems, we use a different model for generating networks with communities. Again, we take as parameters the partition of $n$ nodes into $r$ communities. However, the mechanisms for generating intra- and inter-community edges are now different. We generate every community based on the preferential attachment model by Barabási and Albert (1999), which takes a parameter $m$ that defines the number of connections a new node makes to existing nodes when added to the network. The inter-community edges are then sampled similarly as before by randomly deciding for every pair of nodes in different communities if they should be connected by an edge or not. The probability of connecting two nodes of different communities is based on a third parameter $\rho_{out}$. It is no coincidence that this parameter is called $\rho_{out}$, as the inter-community density on a network generated by this model will on average be $\rho_{out}$. With the described model we will almost always generate a connected network (at least for reasonable values of $\rho_{out}$). Moreover, the network will have a power-law tailed degree distribution if the number of communities is significantly lower than the number of total nodes in the network. The parameter $\rho_{out}$ has to be chosen with care in order to compare different networks with each other because the number of communities and their sizes affect the intra-community density and $\rho_{out}$ has to be selected appropriately to match, for example, a targeted density *ρ* of the network as a whole. Figure 1 shows three networks with three communities each, differing only in the connectivity between communities.

### Agent behaviour

While the previous section discussed the model of *which* agents are influencing which other agents, another important decision to make when creating an agent-based model is *how* the agents interact with each other. Agents in our model behave as they do in the work of Ross et al. (2019). The following section will, therefore, only briefly review the model and the empirical research its assumptions are based on.

The model simulates a simplified scenario of opinion formation. It is assumed that every agent $i$ has an *opinion* $o_{i}$ with values in $\left\{ { + , - } \right\}$, representing either a positive or negative stance regarding a specific topic. Moreover, the opinion is fixed in time, that is, for the whole duration of the simulation an agent always has the same opinion. The opinion is randomly initialized as uniformly across the population, that is, every agent has equal probability of getting $+$ or $-$ as their opinion. This is because the model is not used to study how people change their opinions, but rather how people can become silenced when they feel that their opinion is not adequately represented in the population. The second property of an agent $i$ is its *willingness to self-censor* $\Phi_{i}$ (Hayes et al. 2005, 2010). It determines whether an agent is easily silenced or holds their opinion even in the face of overwhelming opposition. It is also constant in time, as we assume this to be a relatively stable characteristic of a person. In our experiments, the willingness to self-censor is initialized for each agent as a uniformly distributed random value in [0,1].

The next property of an agent is its *confidence* $c_{i} \left( t \right)$. After each step, it is compared to the *willingness to self-censor* $\Phi_{i}$ and, if greater, the agent communicates its opinion to its surroundings (and is called *speaking*), whereas if smaller, the agent is *silenced* and does not speak out its opinion. It changes over the course of a simulation depending on the opinion climate surrounding an agent. Accordingly, the *opinion climate at time* $t,\delta_{i} \left( t \right)$, observed by an agent $i$, is used to update its confidence. It is defined as $\delta_{i} \left( t \right) = \frac{{n_{s} \left( {i,t} \right) - n_{o} \left( {i,t} \right)}}{{n_{s} \left( {i,t} \right) + n_{o} \left( {i,t} \right)}}$, where $n_{s} \left( {i,t} \right)$ is the number of neighbours of agent $i$ openly supporting its opinion, while $n_{o} \left( {i,t} \right)$ is the number of neighbours openly opposing it. There is no change to an agent’s confidence ($\delta_{i} \left( t \right) = 0$) when its neighborhood is completely silent. Confidence is updated as follows: $c_{i} \left( t \right) = 2 \times \left( {1 + e^{{ - \widehat{{c_{i} }}\left( t \right)}} } \right)^{ - 1}$, where $\hat{c}_{i} \left( t \right) = max\left\{ {\hat{c}_{i} \left( {t - 1} \right) + \delta_{i} \left( t \right); 0} \right\}$. The value $\hat{c}_{i} \left( t \right)$ is initialized as a uniformly distributed random value in [0,1]. The transformation into $c_{i} \left( t \right)$ ensures that it stays within this range. As a result of these definitions, if there are more agents in the neighborhood of an agent $i$ expressing their support for the opinion of $i$ than there are agents opposing it, then $\delta_{i}$ will be positive and agent $i$’s confidence increases. Similarly, $\delta_{i}$ is negative if there is more opposition than support in the neighbourhood of agent $i$ and its confidence will drop. It should also be emphasised that only non-silenced neighbours are considered when computing $\delta_{i} \left( t \right)$. Silenced agents have no influence on the opinion climate, which is also why an agent becoming silenced can trigger a cascade of multiple agents becoming silenced or speaking again. In line with previous empirical research, agents with low confidence are more strongly influenced than agents who are already confident (cf. Matthes et al. 2010). This relationship is symmetrical: firm opinions are harder to erode, which can be argued on the basis of cognitive dissonance (Festinger 1957) and selective exposure (Knobloch-Westerwick 2014).

### Experimental design

With the model fixed, it can now be connected to our research questions of how communities affect opinion expression and the formation of a spiral of silence.

In summary, the model has the following parameters:

1. the total number of agents,
2. a randomized initialization method of the *willingness to self-censor* $\Phi_{i}$ for the agents,
3. a randomized initialization method of the *confidence* $\hat{c}_{i} \left( t \right)$ for the agents at time t = 0,
4. the ratio of agents holding the positive opinion to agents holding the negative opinion,
5. the number of communities in the network,
6. the intra- and inter-community density of the communities, controlled by parameters $m$ and $\rho_{out}$ of the network model.

These parameters can be varied to measure how they affect the observable properties of the model over the course of a simulation. Since we aim to study opinion expression, and specifically the emergence of a spiral of silence, we first have to define what we consider a spiral of silence in our model. Again, we take inspiration from Ross et al. (2019), where the ratio of agents expressing their opinion to silenced agents was examined—unsilenced agents were also distinguished into those belonging to the majority or minority opinion, based on all agents expressing their opinion. We say that a spiral of silence occurs in case “most” agents of one of the two opinions become silenced whereas the agents with the other opinion are almost all expressing their opinion.

The following (virtual) experiments consist of varying the model parameters while observing the dependent variables and thus interpreting the relationship between community structure and opinion expression. Note, however, that only the last two of the six model parameters are directly related to community structure while the others are indirect results of the modelling process in the context of the spiral of silence. The parameters 1–4 are therefore simply fixed to sensible values, while we make sure that their choice does not affect the results we obtain when varying parameters 5 and 6 (see the following section). We fix the number of agents to 1000 and initialize the willingness to self-censor and confidence by drawing from a uniform distribution in [0,1] independently for each agent. Each agent is independently assigned either the positive or the negative opinion (with a 50% probability of each). Since the opinion is also assigned independently of community membership, the distribution of positive and negative opinions is close to equal in each of the communities. While communities in real networks often exhibit homophily, and thus agents in the same communities should have more similar opinions, we chose not to model this explicitly as it would make it hard to identify which results are due to the network structure itself, or due to the fact that agents hold more similar opinions if they are in the same community.

Next to decide is how to vary the community-related parameters of the network model to study the research questions. Recall that, specifically, the goal is to examine if and how the fragmentation of a network into communities leads to an increased resilience against a spiral of silence. This can be investigated by varying the number and inter-community density of the communities generated by our network model. Note, however, that Ross et al. (2019) found that increasing the density of the networks, lead to a stronger spiral of silence effect. To account for this effect and study only the influence of the different community structures we have to make sure that the overall density of all generated networks stays constant.

Accordingly, for the first experiment we generate networks with 10 communities and vary the inter-community density $\rho_{out}$ passed to our model. Then we study the relative size of the minority opinion among all agents expressing their opinions. Increasing the inter-community density without also changing the intra-community density, would make the overall network denser, leading to a higher synchronisation and a stronger spiral of silence. To deal with this problem we do not vary the inter-community density directly but change the $m$ parameter of the Barabási-Albert model used to generate every community. The inter-community density is then chosen as a function of $m$ such that the overall density stays constant.

In the second experiment we study the effect of the number and size of communities on the ability of a minority to keep expressing their opinion. To this end, we generate networks evenly partitioned into a varying number of communities (2–10). The overall density of the networks is kept constant by modifying, in this case, the inter-community density accordingly.

### Validation

An important step when working with agent-based models is validation. It is meant to guarantee that a model is an accurate representation of the studied real-world process. The following validation steps are based on the validation frameworks by Sargent (2013) and Klügl (2008) and include manually assessing visual animations of the model, studying degenerate edge cases, ensuring replicability of results in multiple runs, reproducing known results of Ross et al. (2019), and a sensitivity analysis of the input parameters.

**Fig. 2:**
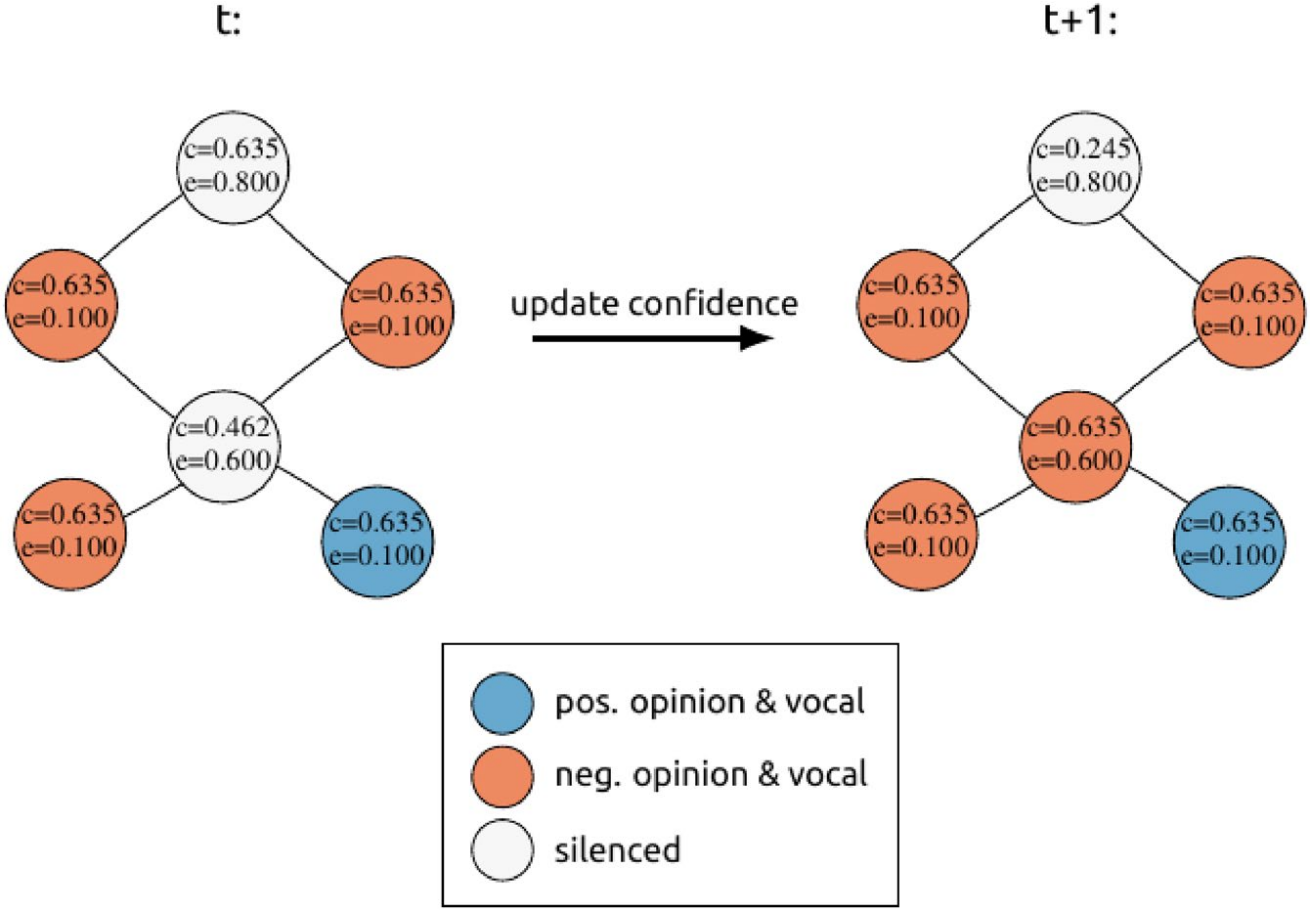
Example confidence update visualisation generated using the program for running the simulations. The c and e values represent an agent’s confidence and willingness to self-censor, respectively. The colors indicate the current state of an agent. In the example, we see the silenced agent in the middle gain confidence because in its neighborhood its opinion (−) is more prevalent than the (+) opinion. As a result of the increased confidence, the agent starts expressing its opinion again

Since our implementation allows for visual inspection during simulation runs, the validation process was started by comparing the agent interactions in very small model instances step by step to the expected behaviour, described in Sect. 3.2 (cf. Fig. 2). We also studied edge cases such as setting the willingness to self-censor to zero and making sure that no agents were ever silenced, or that in a model instance where all agents hold the same opinion, agents would over time all be expressing their opinion and not be silenced.

The experiments in the following section were always run multiple times to check that we had enough runs to get stable statistical results. Since we are relying on the agent behaviour described by Ross et al. (2019), we reproduced some of their results without bot agents by replacing our SBM network model with a simple Barabási–Albert model.

Another means of validation is running a sensitivity analysis, that is, examining if small changes in the model input parameters lead to vastly different outcomes. The underlying motivation is that the real-world model parameters can typically not be quantified perfectly, introducing a variability in the model inputs. This would make a very sensitive model less useful for predicting events in the real world. We used the one-factor-at-a-time (OFAT) method of sensitivity analysis described in (ten Broeke et al. 2016), varying the parameters described in the previous section one at a time while holding the other parameters constant, and validated that any variation in the outcomes was relatively small and that there were no critical points at which the behaviour changed extremely. Naturally, the first parameter, the number of total agents in the model, affected the absolute size of the factions (i.e., agents with positive and negative opinions, speaking or silenced agents). However, the relative sizes stayed more or less the same, except for very small instances of 50 agents or less. Varying the randomized initialization method for the agents’ willingness to self-censor and the confidence (e.g., using uniform distributions in [0,2], [0,5] and [0,10], or exponential distributions with mean 1, 5 and 10) had almost no effect on the final stable state and thus the outcome of the experiments. This seems to be because a few steps into a simulation run, the confidence values adapt based on the values of their surrounding agents, a behaviour that was also observed by Ross et al. (2019).

**Fig. 3:**
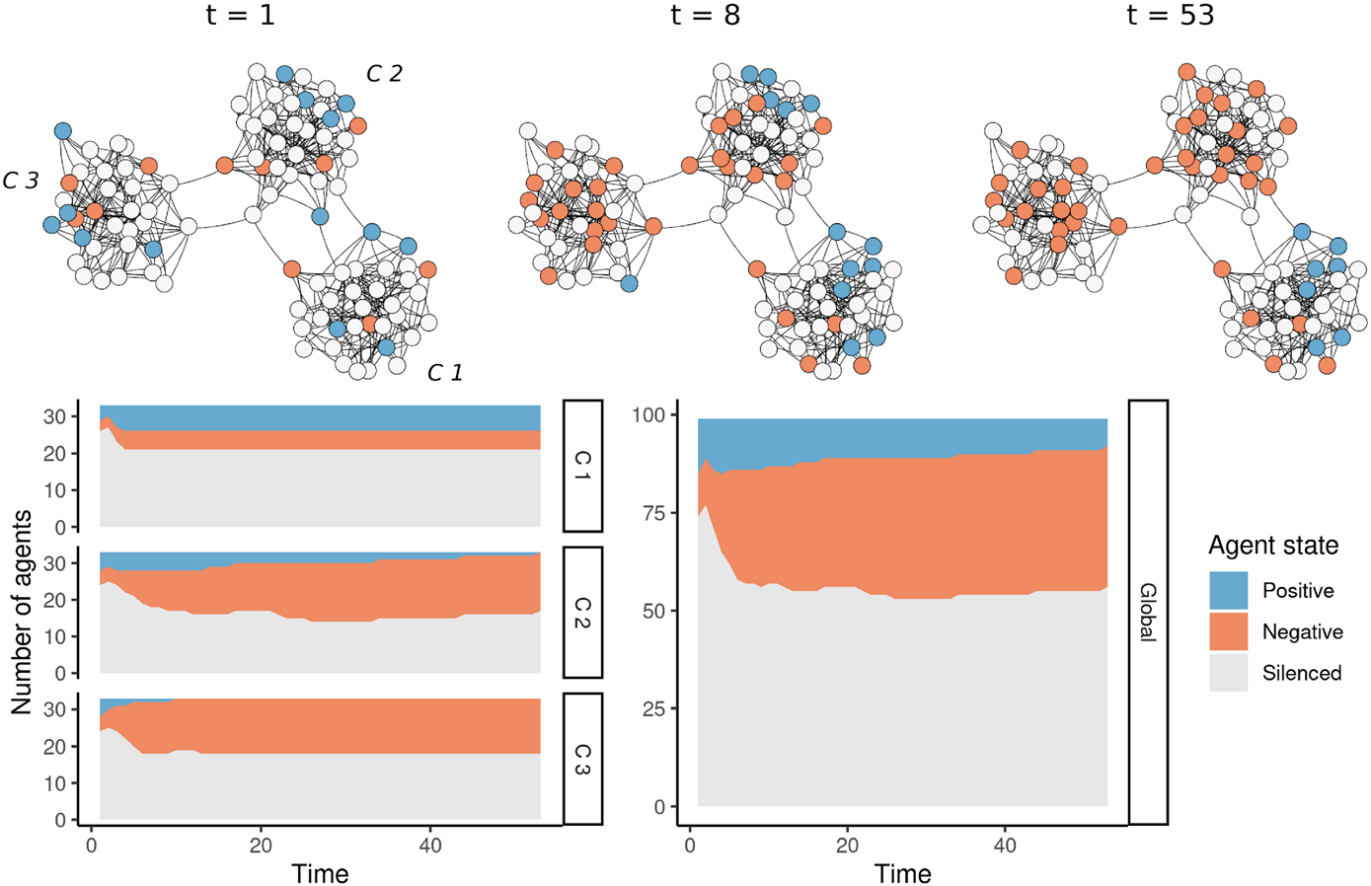
Visualization of a simulation run of a single model instance. At the top, the model’s state at three different points in time is drawn (start, after 8 steps, after the stable state is reached). The stacked area plots at the bottom display the distribution of agent states over time

**Fig. 4:**
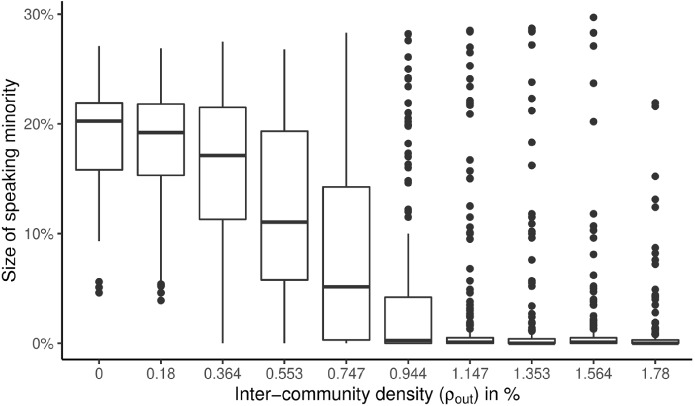
The effect of inter-community density on the ability of the minority to keep expressing their opinion. The horizontal lines inside the boxes represent the median, while the upper and lower boundaries of the boxes are the 25th and 75th percentiles, respectively. The upper and lower whisker extend from the top or bottom of the box to the highest or lowest value, respectively, but no further than 1.5 times the box height. The points represent outliers

The model was most sensitive with respect to changes in the distribution of agents’ opinions. As described above, a 50:50 distribution was used in the experiments, where each agent was equally as likely to hold a positive or negative opinion. When we deviated from this equal distribution of opinions in simulations, we found that it became much harder for the minority opinion not to be silenced, even when there are only loosely connected communities. This is because we initialise agents’ opinions independently across the network and so every community would also reflect a skewed global distribution making it likely that the more frequent opinion dominates in every community. However, while the size of a speaking minority shrinks when the opinion distribution deviates from 50:50, the trend displayed in Figs. 3 and 4 is still visible for distributions up to 30:70, after which the size of the speaking minority becomes essentially zero. We conclude that the results of our model apply in situations where the minority opinion is held by at least roughly 30% of people but caution should be exercised before generalising results to situations with smaller minorities.

The parameters 5 and 6, related to the community structure of the networks, are varied as part of answering the research questions and the results are described in the next section.

Finally, note that Ross et al. (2019) observed that the overall density of the network affects the strength of the observed spiral of silence. In a dense network, the high connectivity between agents seems to foster quick synchronization and no minority opinions are expressed anymore. As a result, and as already mentioned in the previous section, the overall network density was held constant when varying the community structure of the networks.

The external validity of a model is the ability that model results directly translate to scenarios observed in the real-world. In a best-case scenario, external validity can be tested by letting the model reproduce known empirical findings in the domain of interest, this is sometimes called predictive validity (Sargent 2013). To validate the present study, however, such empirical research would likely involve a large-scale survey asking participants for their opinions on a particular topic and asking if they are expressing that opinion publicly. Moreover, since we explicitly focus on the effect of community structure on the spiral of silence a comparable empirical study would also have to study multiple communities, and in the best case also quantify their mutual influences. There are various survey studies on the spiral of silence, see for example Glynn et al. (1997) for an overview. However, these are mainly concerned with verifying that the main mechanism of the spiral of silence actually exists, namely people self-censoring in face of a perceived opposing opinion climate. While there are some studies on the spiral of silence that explicitly mention communities (Salwen et al. 1994; Carter Olson and LaPoe 2017), they mostly focus on few, separate communities and not the interaction between multiple of them.

## Results

Before we present the results of the experiments discussed in the previous section, we would like to give a better intuition on how the different modelling decisions work together. To this end, Fig. 3 visualises the simulation of a single model instance from time $t = 1$ to when the stable state is reached. The model consists of 99 agents uniformly distributed among 3 communities connected to each other only by a few connections. Initially, both opinions are expressed more or less equally in all communities. Over time, however, in communities “C 2” and “C 3” the negative opinion starts to dominate while any agents with positive opinions become silenced. In “C 1”, a stable state, with some agents expressing positive and some expressing negative opinions, is reached. By the end, more than half of the agents are silenced. Because “C 2” and “C 3” are dominated by agents expressing negative opinions, the global distribution of expressed opinions is also heavily favoured towards negative opinions. The following experiments examine this behaviour for larger instances, with varying model properties, and averaged over a large number of runs.

Figure 4 shows the results for the first experiment, where the network contained $r = 10$ equally-sized communities, and the parameter $m$ of the Barabási–Albert model was varied in $\left\{ {1,...,10} \right\}$, while keeping the overall density of the networks on average constant at $\rho \approx 1.8\%$. This implied the corresponding variation of inter-community density displayed on the x-axis. The y-axis shows the percentage of agents that openly express the minority opinion, i.e., the opinion openly expressed by fewer agents compared to the other opinion. The displayed plot visualizes results of 500 randomized runs per configuration, 5000 runs overall.

We omit the percentage of agents expressing the majority opinion as well as silenced agents because the size of silenced agents stayed relatively constant and every increase in the majority opinion is reflected as a decrease in the minority opinion.

As expected, the speaking minority is strongest for $\rho_{out} = 0$, with 20% of the minority opinion still expressing their opinion. This is unsurprising because for $\rho_{out} = 0$ the communities are disconnected and the spiral of silence process develops separately for each community. For 10 communities there is a high chance that there are communities in which the global minority opinion is dominating and not silenced. Since the communities are not connected to each other, such local majorities will not be silenced and are registered as part of the global minority expressing their opinion. This effect of completely disconnected communities seems to wear off when approaching an inter-community density of 1%. Further increasing the inter-community density, the ratio of the speaking minority stabilises at 1.5%, a value close to the one reported by Ross et al. (2019) for a network without communities at approximately $m = 6$. This to be expected, as the overall density in our networks matches a density in a network without communities generated only by the Barabási–Albert model for $m$ between 5 and 6.

**Fig. 5:**
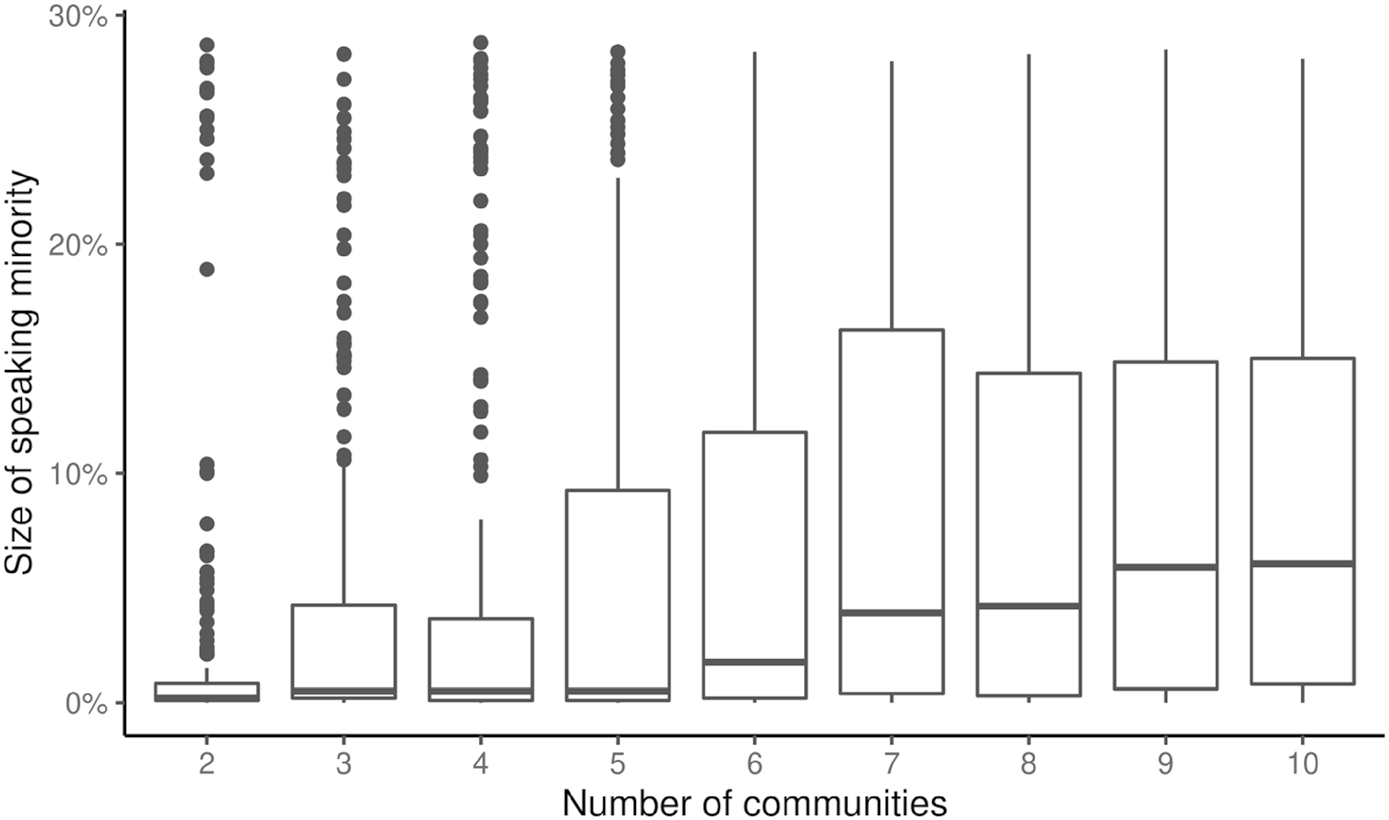
The effect of the number of communities on the ability of a minority to keep expressing their opinion. The boxplot representation is the same as in Fig. 3

Figure 5 shows the result of the second experiment. Here, the number of equally-sized communities $r$ was varied in $\left\{ {2,...,10} \right\}$, while keeping the overall density of the networks on average constant. The parameter of the Barabási–Albert model was fixed at $m = 5$. The displayed boxplot visualizes results of 500 randomized runs per configuration, 5000 runs overall.

Intuitively a network with fewer, but larger communities should behave more similarly to a network without communities than a network fragmented into many smaller communities. Random disbalances of the agents’ properties in a community can influence the dominating opinion in that community. In case of more communities, the probability that there will be communities with a differing minority/majority opinion compared to the overall network are higher.

From Fig. 5 it is apparent that a higher fragmentation into more, but smaller communities leads to more agents expressing a minority opinion. In the case of two communities, on average only 2.5% of agents belonging to the minority opinion were still expressing their opinion at the end of the runs. When the networks consist of 10 equally-sized communities, up to 10% of agents holding the minority opinion are still openly expressing it. Together with the fact that the overall density of the network was kept constant, this seems to indicate that a fragmentation into more, smaller communities is beneficial for minorities to keep expressing their opinions and not be silenced by the majority.

## Discussion

The present study examines how community structure affects the formation of public opinion, following the assumptions of the spiral of silence theory.

As a first result, we find that a high number of relatively small communities leads to a situation in which the minority opinion is still expressed by a larger part of the total population, compared to a scenario with a small number of large communities. In the former situation, entire subcommunities exist which have “local” majority opinions, undeterred by the fact that the global consensus is the opposite. Whether one views these small subcommunities in a positive light, as safe spaces in which minority opinions are still allowed to flourish, or negatively, as echo chambers of radicalisation, is open to interpretation. In the context of market segments, this result explains situations in which some markets lose interest in a product, as may happen in the airline industry if the flight shame movement continues to grow in the Western cultural sphere. Central nodes that are connected to many individuals have a greater influence on the opinions of others (van Eck et al. 2011). Furthermore, the influence of opinion leaders in a political context could be shown in the study of Twitter communities on the 2016 U.S. presidential election of Clinton and Trump, where certain opinion leaders led to a political homogeneity of the communication of communities (Guo et al. 2020). Thus it can be argued that our results of these small communities might be led by opinion leaders and their minority opinions. As Wu et al. (2015) reported, the frequency of connections to other nodes can lead to a convergence between communities, but this requires a uniform activation of all users and not only those users who exist as interfaces between the communities. Due to the fact that the connections to the individual communities are dependent on a few agents, they may not be in close contact with the opinion leaders and therefore are unlikely to be influenced (Liu 2007).

The second key result is that the more interconnected these communities are, the more likely a “global” spiral of silence is to emerge again. If the division of the network into communities creates “safe spaces” for minority opinions, a high degree of interconnectedness negates this effect. In other words, the more consumers from different markets communicate with each other, the more likely a spiral of silence is to emerge on a global scale. According to a related result by Sohn (2019), such a “global” spiral is also likely to occur in the case of mass media spreading a homogeneous opinion to a large part of the population. In an age of increasing global interconnectedness, in which information technology allows consumers to post their opinions on the internet for the entire world to see, this result would seem to predict an increasing homogenisation of consumer opinion. However, “global” here refers to a spiral of silence encompassing the entire network of, in this case, 1000 actors. As Sohn (2019) points out, a truly world-wide spiral of silence is unlikely to occur, since the social network in neither simulation should be seen as an approximation of the social network of the 7.7 billion people in the world population, but rather the social network of some population of interest.

Several other studies reported results that are comparable to our findings. Wu et al. (2015) investigated different network topologies, one of which consisted of a network split into two communities. Similar to us, they found that “the number of silencers grows as the degree of coupling increases”. However, while we seem to replicate some of their results, they used a very different agent behaviour to simulate the spiral of silence process. In particular, they chose a single agent as the source of the initial opinion propagation then spreading to the rest of the network, and introduced an “immunity” that can keep agents from being silenced. The survival of minority opinions in the presence of sufficient modularity (i.e., community structure) is also a central result of Banisch and Olbrich (2019)’s model. Their approach shares with ours the distinction between opinion and opinion expression and it also relies on a positive/negative feedback mechanism not unlike those found in the spiral of silence theory, where agents are reinforced (or not) in their opinions by those around them. However, in Banisch and Olbrich’s model, agents are selected uniformly at random from the population and forced to express their opinions; silence is not an option. Since this is one of the defining features of the spiral of silence theory, Banisch and Olbrich’s results, while similar to ours, are the consequence of fundamentally different assumptions. In a direct comparison with both Wu et al. (2015) and Banisch and Olbrich (2019), the contribution of our research is to show that our model of the spiral of silence theory provides an alternative explanation for similar results.

When interpreting the results, the spiral of silence model needs to be distinguished from other models where the similarities are more superficial. The classical bounded confidence models such as the Deffuant model and the Hegselmann and Krause model show how opinions change over time in a continuous opinion value, but they do not show how confident agents feel about expressing their opinions and are therefore not convenient for modelling the processes of the spiral of silence. In the opinion dynamics model of Ye et al. (2019), which was inspired by the Friedkin-Johnsen model, a discussion process is simulated in which individuals adjust their private and expressed opinions in the network through the social influence of peer pressure. Here, variables such as stubbornness, resilience, individuals' opinions are taken into account, which in a very dense network leads to quickly reach a "steady-state of persistent disagreement". However, these results are difficult to compare with our current study, since communities are not explicitly considered and the simulations focus on smaller numbers of agents, in contrast to our goal of simulating opinion dynamics in the large-scale online context. Although the results of Stern and Livan (2021) do not shed light on the spiral of silence, they do provide insights into opinion dynamics and show how opinions are distributed among communities in the network when they are created using the stochastic block model. The results of the study show that it is more difficult for networks with closed communities to reach a common consensus when many different opinions exist, although the conceptualisation of what constitutes an opinion is rather different in their model and it lacks the distinction between opinion and opinion expression.

In terms of practical implications, companies can learn from the findings of this study. As described in Sect. 2, online opinion formation is a crucial factor for business success. Analysing the potential impact of community size, number and inter-connectedness reveal several implications for strategic decision making within a company. For instance, establishing distinct communities for specific target markets, such as countries or products, could reduce the danger of fast-spreading negative opinions in case of an evolving corporate crisis. The management and interaction with customers can be used to establish partnerships with users of distinct communities, leading to more control on discussed topics on social media (Etter and Vestergaard 2015). In this context, the silence of a company on a discussed topic can have a negative impact on the opinion climate, and thus, on the business success (Stieglitz et al. 2019). Furthermore, the findings suggest that several smaller communities could act as a stabiliser for minority opinion expression. In the context of a corporate crisis, the minority expresses a positive opinion. Thus, the companies could maintain a positive opinion in those specific target markets. However, the establishment of distinct target markets, and therefore, communities, may not be sufficient enough in order to secure business success. Thus, the findings of this study implicate that companies should actively (1) observe and (2) manage, and (3) maintain the individual communities. Therefore, online community management may play a central role in a company’s marketing planning. As a first step, potential communities need to be identified and continuously observed. Second, those communities should be actively managed, to this end, the company should communicate to customers and react to their feedback (e.g., customer co-creation). Third, the company should try to maintain a positive online opinion within the community by considering step two. To this end, the company might place corporate opinion leaders within the communities as communicators.

Of course, this study also faces distinct limitations. On the one hand, limitations of the spiral of silence theory have to be considered. Thus, the study models changes in the willingness to express one's opinion and not shifts in the held opinions themselves. On the other hand, the applied model is suitable for topics on which people have already formed their opinion and which do not change so quickly. Since the model gives each node in the network a 50% chance of being of the positive opinion, and a 50% chance of the negative opinions, the initial distribution of opinions within each community will rarely be exactly 50–50, but approach this in the long run. Such an approach is inappropriate to model a setting in which communities differ ideologically, such as an online community of car enthusiasts and an online community of environmentalists. However, in regard to realms such as general products or brand images, the applied model does allow concrete deductions for research and practice. Another limitation of our research is the empirical validation of the output of the model, considering that we do not have comparisons of theoretical foundations that deal with the spiral of silence theory linked to community structures. This problem of missing and non-existent data has already been addressed in previous research (Fagiolo et al. 2007; Klügl 2008). For this reason, we took the approach of empirical data as input reference (Waldherr and Wettstein 2019), taking into account the empirical findings during the development of the model and their parameter settings. As Alvarez-Galvez (2016) indicates, using a connection of multiple techniques and data (real networks, media information, and survey methods), these agent-based models might be validated further in future research in order to gain a better understanding of the processes of opinion formation and their dynamics at different levels, beyond our validation efforts described in Sect. 3.4.

Analysing the findings provides foundations for several possible areas of future research. This could result in further insights about fields of application in which communities differ ideologically. Moreover, future research might distinguish between different types of actors within the network. Especially in the context of business success, actors such as opinion leaders and corporate influencers might play a special role. Therefore, the impact of opinion leaders, which may influence more or fewer people in relation to other actors, on global and community based spiralling effects could be examined.
